# Distinctive mitochondrial genome of Calanoid copepod *Calanus sinicus *with multiple large non-coding regions and reshuffled gene order: Useful molecular markers for phylogenetic and population studies

**DOI:** 10.1186/1471-2164-12-73

**Published:** 2011-01-27

**Authors:** Wang Minxiao, Sun Song, Li Chaolun, Shen Xin

**Affiliations:** 1 KLMEES and JBMERS, Institute of Oceanology, Chinese Academy of Sciences, 7 Nanhai Road, Qingdao 266071, China; 2 Graduate University, Chinese Academy of Sciences, 19 Yuquan Road, Beijing 100039, China; 3 Huaihai Institute of Technology, 59 Cangwu Road, Lianyungang 222005, China

## Abstract

**Background:**

Copepods are highly diverse and abundant, resulting in extensive ecological radiation in marine ecosystems. *Calanus sinicus *dominates continental shelf waters in the northwest Pacific Ocean and plays an important role in the local ecosystem by linking primary production to higher trophic levels. A lack of effective molecular markers has hindered phylogenetic and population genetic studies concerning copepods. As they are genome-level informative, mitochondrial DNA sequences can be used as markers for population genetic studies and phylogenetic studies.

**Results:**

The mitochondrial genome of *C. sinicus *is distinct from other arthropods owing to the concurrence of multiple non-coding regions and a reshuffled gene arrangement. Further particularities in the mitogenome of *C. sinicus *include low A + T-content, symmetrical nucleotide composition between strands, abbreviated stop codons for several PCGs and extended lengths of the genes *atp6 *and *atp8 *relative to other copepods. The monophyletic Copepoda should be placed within the Vericrustacea. The close affinity between Cyclopoida and Poecilostomatoida suggests reassigning the latter as subordinate to the former. Monophyly of Maxillopoda is rejected. Within the alignment of 11 *C. sinicus *mitogenomes, there are 397 variable sites harbouring three 'hotspot' variable sites and three microsatellite loci.

**Conclusion:**

The occurrence of the *circular subgenomic fragment *during laboratory assays suggests that special caution should be taken when sequencing mitogenomes using long PCR. Such a phenomenon may provide additional evidence of mitochondrial DNA recombination, which appears to have been a prerequisite for shaping the present mitochondrial profile of *C. sinicus *during its evolution. The lack of synapomorphic gene arrangements among copepods has cast doubt on the utility of gene order as a useful molecular marker for deep phylogenetic analysis. However, mitochondrial genomic sequences have been valuable markers for resolving phylogenetic issues concerning copepods. The variable site maps of *C. sinicus *mitogenomes provide a solid foundation for population genetic studies.

## Background

Copepods play an important role in the aquatic ecosystem and are highly diverse. They comprise a multitude of taxa including 200 families, 1,650 genera and 11,500 species [1], although this estimation may represent only 15% of the actual number [2]. Copepods have successfully colonized almost all aquatic regimes and have developed diverse life styles [3]. Therefore, phylogenetic studies are required to develop a complete biodiversity inventory of the group, which will enable the question of how copepods have acquired such diversity over time to be investigated.

Several incompatible classification schemes have been proposed for copepods on the basis of morphological characteristics [4]. Since the incorporation of copepods as a monophyletic group in 1859, phylogenetic studies have focused on the natural relationships between the incorporated orders, Calanoida, Cyclopoida, Gellyelloida, Harpacticoida, Misophrioida, Monstrilloida, Mormonilloida, Platycopioida, Poecilostomatoida and Siphonostomatoida [3]. Dussart (1984) classified Calanoida and Poecilostomatoida together in the lineage Cyclopinidae-Oithonidae-(Poecilostomatoida-Calanoida) [5] while other researchers have classified the Calanoida outside Podoplea, at the relative basal position [3,6]. Kabata, Marcotte and Boxshall hypothesised that Poecilostomatoida is the sister group to Cyclopoida. However, other studies have placed Poecilostomatoida and Siphonostomatoida within close phylogenetic affinity [3,6]. Recently, Boxshall reassigned Poecilostomatoida as a suborder of Cyclopoida [7]. The relationships among copepods and other subgroups of Pancrustacea have yet to be elucidated with 11 alternative sister group hypotheses being proposed for the taxon [8]. The recent ambiguous status of copepod phylogenetic research is due at least in part to the limited diagnostic morphological characteristics, difficulty in accessing morphological homology and a poor fossil record.

In metazoans, the mitochondrial genome is usually a circular, double-stranded DNA molecule (mtDNA), which spans a general length of 16 kb but can vary from 14 to 48 kb. The gene content is conserved with 37 genes: 13 protein-encoding genes, two ribosomal RNA genes, 22 transfer RNA (tRNA) genes and one or more non-coding region(s) containing signals for transcription and replication of the mtDNA [9]. Several advantages including accelerated substitution rates, (almost) unambiguous orthology and being genome-level informative [10,11] have allowed the mitochondrial genome to be widely used for population studies [12,13], phylogeography [12,14] and phylogenetic relationships at various taxonomic levels across animal taxa, particularly in arthropods [15-17]. Furthermore, extensive intraspecific polymorphism in the non-coding regions facilitates studies at population level [17]. However, there is little information concerning the structure and genetic polymorphism of the non-coding regions in crustaceans.

Despite the vast diversity of copepods, few mitochondrial genomes have been charted. Taxon sampling has been biased to certain orders including Harpacticoida: *Tigriopus japonicus *[18,19], *Tigriopus californicus *[14]; Siphonostomatoida: *Lepeophtheirus salmonis *[20] and Cyclopoida: *Paracyclopina nana *[21]. More mitochondrial genomes with increased taxon coverage are required to resolve several issues concerning copepod phylogeny including its phylogenetic position within Pancrustacea and the relationship of its component orders. *Calanus sinicus *(Copepoda: Calanoida) dominates continental shelf waters in the northwest Pacific Ocean, linking primary production and the larvae and juveniles of fishes [22]. Given its ecological importance, *C. sinicus *is one of the target species in the China-GLOBEC program. Despite this status, there is little information concerning the population genetics of this species owing to the lack of suitable genetic markers. This study presents a near complete mitochondrial genome of *C. sinicus*, which represents the first member of the Calanoida. The gene order of *C. sinicus *was compared with other copepods to identify the evolution of the mitochondrial genomes in this group. Combining the new mitogenome and previously published mitogenomes from arthropods, a preliminary phylogenetic analysis was carried out to investigate the relationships between several orders in Copepoda and their positions within Pancrustacea. In addition, intraspecific polymorphisms of major loci in 11 *C. sinicus *mitogenomes from four populations were compared to screen potential markers for population studies.

## Results and Discussion

### Genome Organization

A long-PCR-based genome sequencing protocol was adopted for animal mtDNAs. However, this technique failed to amplify a fragment containing partial non-coding regions and two tRNA genes. Several unknown factors including gene rearrangement, notable base composition bias, an extended length of GC-rich tract, highly repeated regions and stable secondary structures could terminate the movement of the polymerase and therefore complicate the recovery of mitogenomes from copepods using this technique [19,21,23].

**Figure 1:**
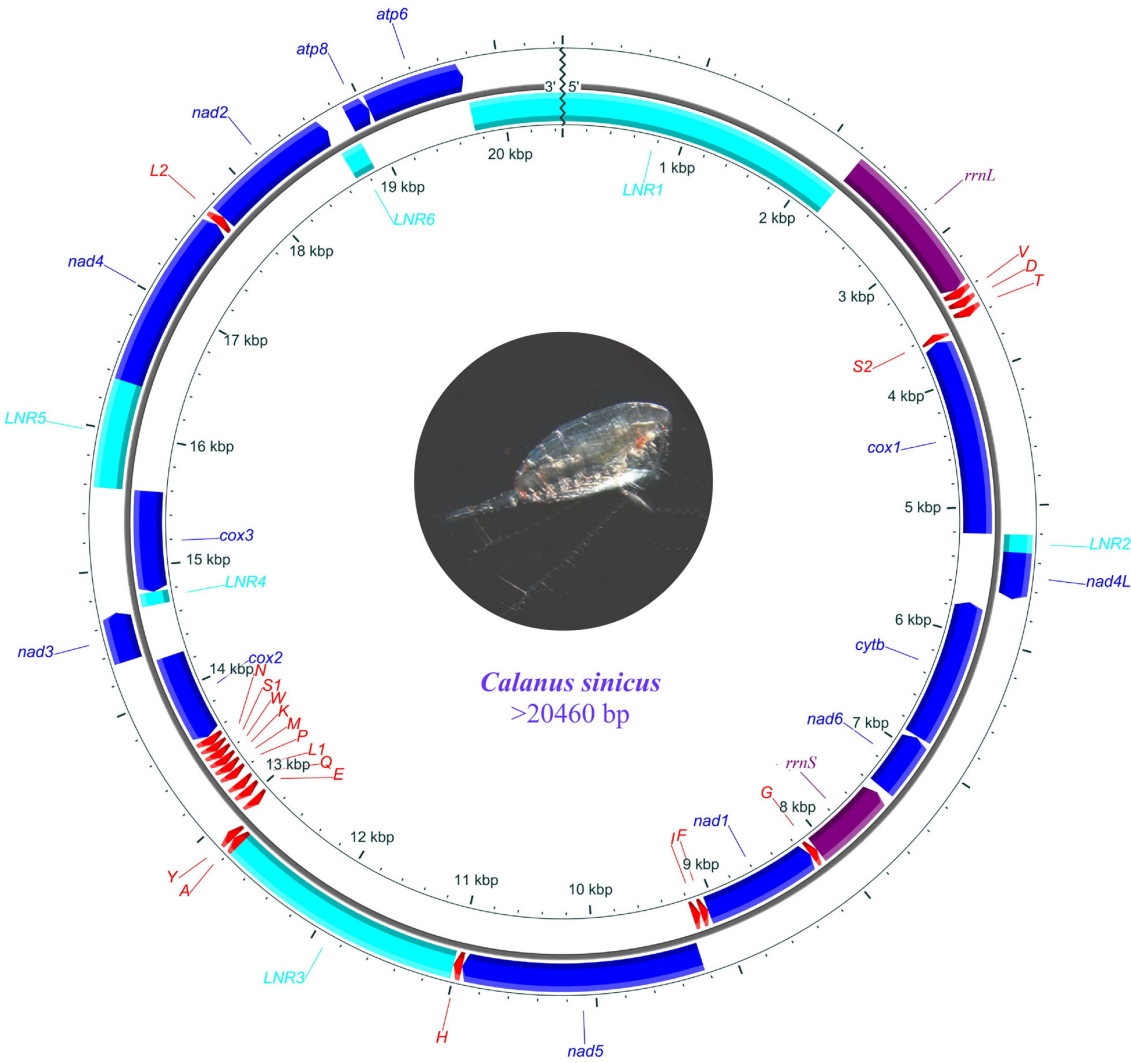
**Mitochondrial genome organization of Calanoida copepod *Calanus sinicus***. direction of gene transcription is indicated by the arrows. Protein-coding genes are shown as blue arrows, rRNA genes as purple arrows, tRNA genes as red arrows and large non-coding regions (>100 bp) as cyan rectangles. tRNA genes are labelled by single-letter IUPAC-IUB abbreviations (L1: CUN; L2:UUR; S1:AGN; S2:UCN) while other genes are represented as outlined in the abbreviations section. Ticks in the inner cycle indicate the sequence length.

The 20,460 bp assembled contig (Figure 1, Table 1) comprised all but two tRNA genes (*trnR *and *trnC*), and included 13 protein coding genes (*cox1*-*3*, *nad1*-*6*, *nad4L*, *atp6*, *atp8 *and *cytb*), two rRNA genes (*rrnS *and *rrnL*) and 20 tRNA genes. In addition, one of the long non-coding regions (LNR) between *trnH *and *trnA *was proposed as a control region (CR) on the basis of the secondary structure motifs identified. The majority of metazoan mitogenomes contain two abutting gene blocks, *nad4*/*nad4L *and *rrnS*/*rrnL*. However, these are separated in copepods. The 35 genes were located in three clusters interleaved by long non-coding regions (LNR1, LNR3 and LNR5). Unlike *Tigriopus sp*. [14], mitochondrial genes were encoded on both strands in *C. sinicus*, and the minority (*rrnL*, *trnV*, *trnD*, *trnT*, *nad4L*, *nad5*, *trnH*, *trnA*, *trnY*, *nad3*, *nad4*, *trnK*, *nad2*, *atp8 *and *atp6*) were identified on the H-strand (as defined by molecular weight). Of the 20 tRNA genes, 17 were arranged in three main clusters, V-D-T-S_2_, F-I and A-F-Y-E-Q-L_1_-P-M-K-W-S_1_-N, reading clockwise. Compactness is a characteristic feature of mitochondrial genomes [10] and there were small gene overlaps at three gene borders. The largest overlap was identified between *trnY *and *trnE*, with a length of five nucleotides.

**Table 1:** Mitochondrial genome profile and nucleotide composition of *C. sinicus*.

Feature	strand	Position	Length	Start	Stop	AT %	GC-skew	AT-skew	**intergenic nt**^**2**^
***rrnL***^*3*^	H	2244 - 3382	1139			71.7	0.0567	0.0126	>2959(LNR1)
***trnV***	H	3383 - 3447	65			83.1	-0.0888	0.0734	3
***trnD***	H	3457 - 3518	62			80.6	0.165	0.000	9
***trnT***	H	3531 - 3593	63			74.6	-0.126	-0.0643	12
***trnS2***	L	3594 - 3650	57			73.7	0.202	0.0475	0
***cox1***	L	3660 - 5207	1548	ATA	TAA	59.0	0.0439	-0.190	9
***nad4L***	H	5336 - 5671	336	ATA	TAG	61.9	0.0919	-0.202	128(LNR2)
***cytb***	L	5751 - 6887	1137	ATG	TAA	60.2	-0.0050	-0.249	79
***nad6***	L	6898 - 7377	480	ATT	TAG	62.3	0.0822	-0.149	10
***rrnS***^*3*^	L	7429 - 8082	654			73.1	0.264	0.0369	51
***trnG***	L	8083 - 8146	64			87.6	0.000	0.000	3
***nad1***	L	8147 - 9063	917	ATA	TA^1^	62.5	0.000	-0.174	0
***trnF***	L	9064 - 9126	63			66.6	0.237	0.000	0
***trnI***	L	9131 - 9193	63			58.7	0.153	0.0801	4
***nad5***	H	9231 - 10954	1724	ATT	TA^1^	59.5	0.0272	-0.109	37
***trnH***	H	10955 - 11017	63			69.8	0.369	0.0917	0
***trnA***	H	12788 - 12851	64			67.2	0.0488	-0.164	1770(LNR3)
***trnY***	H	12852 - 12912	61			60.6	0.000	-0.0264	0
***trnE***	L	12908 - 12971	64			68.7	0.000	0.0451	-5
***trnQ***	L	13002 - 13067	66			77.5	0.604	-0.102	30
***trnL1***	L	13080 - 13143	64			80.1	0.431	0.104	12
***trnP***	L	13178 - 13240	63			76.2	0.202	-0.0420	34
***trnM***	L	13246 - 13309	64			68.7	-0.0990	0.0917	5
***trnK***	L	13312 - 13374	63			68.2	-0.101	0.0235	2
***trnW***	L	13376 - 13439	64			78.1	-0.142	0.0807	1
***trnS1***	L	13439 - 13498	60			77.0	-0.144	-0.0208	-1
***trnN***	L	13498 - 13565	68			61.8	-0.154	0.0485	-1
***cox2***	L	13571 - 14275	705	ATT	TAA	62.6	0.0749	-0.166	5
***nad3***	H	14338 - 14691	354	ATT	TAA	61.3	0.183	-0.318	62
***cox3***	L	14794 - 15585	792	ATG	TAA	57.1	0.0536	-0.208	102(LNR4)
***nad4***	H	16357 - 17658	1302	ATA	TAA	59.9	-0.0697	-0.142	771(LNR5)
***trnL2***	H	17663 - 17728	66			69.7	0.102	0.174	4
***nad2***	H	17729 - 18697	969	ATA	TAA	61.9	-0.0761	-0.202	0
***atp8***	H	18870 - 19031	162	ATT	TAA	70.4	-0.0811	-0.122	172(LNR6)
***atp6***	H	19034 - 19744	711	ATG	TAG	59.5	-0.0963	-0.126	2

Genes were labelled as outlined in the abbreviations section. AT skew = (A% - T%)/(A% + T%); GC skew = (G% - C%)/(C% + G%).

^1 ^truncated stop codon, which is possibly completed via post-transcriptional adenylation;

^2 ^unassigned nucleotides (positive values) or overlapped nucleotides (negative values) between two adjacent genes with large non-coding regions outlined;

^3 ^initiation or termination positions of ribosomal RNAs defined by adjacent gene boundaries.

### Base Composition and Codon Usage

The H-strand in the *C. sinicus *mitogenome comprises 32.1% A, 19.1% C, 19.3% G and 29.6% T. As presented in Table 1, the overall A + T content of *C. sinicus *is relatively low (61.7%) in comparison with other crustaceans, but within the range for copepods, a minimum of 60.4% in *T. japonicus *to a maximum of 70.8% in *P. nana *(Additional file 1). The same trend was observed in the protein coding genes (PCGs, 60.3%) and non-coding sequences (58.2%), which were lower than those in the majority of crustaceans. The A + T content of structural RNA genes was much richer, being 72.3% and 72.2% for tRNA and rRNA genes, respectively, which is comparable with other crustaceans.

Metazoan mitogenomes normally bear a clear strand asymmetry in terms of nucleotide composition owing to asymmetric deamination of A and C nucleotides on each strand during replication and/or transcription processes [24]. However, there are approximately equal numbers of each complementary nucleotide pair in *C. sinicus*. When measured as AT- and GC-skews ((A%-T%)/(A% + T%) and (G%-C%)/(G% + C%)), the result is close to equality (0.00521) for the former and only moderately positive (0.0405) for the latter. Similar results have been reported in other copepods (Additional file 1; *P. nana*: AT-skew = -0.0457, GC-skew = 0.0598; *S. polycolpus*: AT-skew = -0.0389, GC-skew = 0.0102). In contrast to the whole genome, an anti-A skewness was apparent for all PCGs (-0.177; with a range between -0.318 for *nad3 *to -0.109 for *nad5*) regardless of the strand on which they were encoded, while adenines were slightly preferred in all rRNA genes (0.0125 for *rrnL *and 0.0369 for *rrnS*). As demonstrated in Table 1 and Additional file 1, over-representation of guanines emerges in rRNA and tRNA genes. PCGs represent either neutral (*cytb *and *nad1*), negative (*nad4*, *nad2*, *atp8 *and *atp6*) or positive GC-skewness. Interestingly, all negative GC-skewed genes clustered in a gene block carrying the same transcriptional polarization, possibly because of an inversion or transcriptional polarization of the gene block.

**Figure 2:**
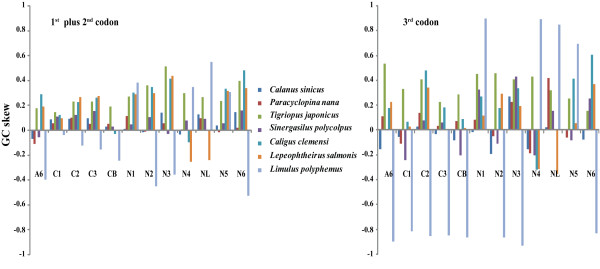
**Strand compositional asymmetry in Copepoda**. GC skewness was calculated for 1^st ^plus 2^nd ^(on the left) and 3^rd ^(on the right) codon positions for the 13 protein-coding genes of Copepoda. For each plot, the values for *Limulus polyphemus *are given. Genes were abbreviated as outlined in the abbreviations section.

To elucidate possible mechanisms that have shaped present-day nucleotide compositional strand asymmetry in the lineage, the GC-skewnesses for individual PCGs of copepods were compared with those of *Limulus polyphemus *(Figure 2). The strand asymmetric profiles of Copepods differed significantly from those of *L. polyphemus *in most PCGs. This suggests a global reversal of the skewness as a possible synapomorphy in the group, probably due to an ancestral inversion of the control region. However, specific nucleotide asymmetric profiles can be identified in all genes with the exception of *cox2 *and *nad3*, possibly because of a shift in the transcriptional polarization of the genes. The 3^rd ^position of the PCGs is less constrained, and they tend to accumulate nucleotide skewness more quickly, making them more likely to be at equilibrium. The opposite results for the skewness at different codons in several genes could be evidence for their recent inversions. Therefore, a complex series of rearrangement events may have occurred in this lineage.

The pattern of codon usage in the *C. sinicus *mitogenome was studied (Additional file 2). A preference for AT-rich codons was identified in *C. sinicus*, as is the case in mitochondrial PCGs of other arthropods. For example, the most frequently used codons are UUU(F) (63 codons per 1000 codons), followed by AUU(I) (54 codons per 1000 codons) and then AUA(M) (49 codons per 1000 codons). Among copepods, the A + T content of the overall mitogenome is highly correlated with the corresponding values in degenerate synonymous sites of protein coding genes (R^2 ^= 0.9918). The A + T-content of the 3^rd ^codon positions (62.6%) in *C. sinicus*, which is only slightly higher than that in *T. japonicus*, is lower than that in most other crustaceans.

### Protein coding genes

More than one reasonable start codon can be predicted for several genes. Therefore, start codons were selected from the candidates following criteria to avoid large overlaps between genes and to keep a conserved length with other crustaceans. There are a total of 11,137 nucleotides encoding 13 protein coding genes in *C. sinicus*, which are at least 147 nt longer than other copepod mitogenomes. The genes *atp6 *and *atp8 *are heavily truncated in other copepods but maintain the regular size in *C. sinicus*, predominantly contributing to the elongation of mitochondrial PCGs. Each of the 13 protein-coding genes in *C. sinicus *start with a typical initiation codon ATD: ATA for *cox1*, *nad1*, *nad2*, *nad4 *and *nad4L*, ATT for *atp8*, *cox2*, *nad3 *and *nad5*-*6*, and ATG for the remainder. Previous studies have reported several atypical initiation codons for *cox1 *in arthropods [25]. However, the copepods studied to date possess a regular start codon (ATA) for *cox1*.

The majority of the 13 protein coding genes terminate with the conventional stop codons TAG or TAA, but *nad1 *and *nad5 *have truncated stop codons (TA) adjacent to a downstream tRNA gene. The presence of incomplete stop codons is common in metazoan mitogenomes, and the shortened stop codons are likely to be completed via post-transcriptional polyadenylation [26].

In view of the clear saturated mutation at the nucleotide level, the amino acids of PCGs were compared among copepods. As illustrated in Table 2, the overall amino acid divergences among the copepods was particularly high, ranging from 0.238 in *cox1 *to 0.768 in *nad4L*. In general, genes encoding proteins for complex I (*nad1*-*6*, *nad4L*) of the electron transport chain were less conserved than others. Altered mutation rates and relatively relaxed selective constraints [27] are the two possible factors responsible for elevated divergence in mitochondrial genes for complex I. However, NADH genes are dispersed within mitogenomes of copepods. Therefore, it is unlikely that several NADH genes would possess altered mutation rates with the same trend, simultaneously. The latter interpretation seems most plausible. Structural or functional constraints at the protein level can lead to locus-specific selective pressures acting on mitochondrial genomes, giving rise to a higher divergence in some PCGs.

**Table 2:** Genetic divergence of the mitochondrial genes among five copepods and 11 individuals of *C. sinicus*.

*gene*	*DB*	*ωB*	*DW*	*ωW*
***atp6***	0.557	0.0736	0.00102	0.157
***atp8***	0.519	NA^1^	0.00314	0.000
***cox1***	0.238	0.0283	0.00115	0.000
***cox2***	0.488	0.0369	0.00098	0.885
***cox3***	0.372	0.0074	0.00211	0.000
***cytb***	0.395	0.0026	0.00070	0.000
***nad1***	0.528	0.0926	0.00115	0.159
***nad2***	0.753	0.0100	0.00255	0.222
***nad3***	0.581	0.0923	0.00103	0.358
***nad4***	0.668	0.0534	0.00184	0.914
***nad4L***	0.768	0.0617	0.000	0.000
***nad5***	0.625	0.0306	0.00173	0.279
***nad6***	0.732	0.0087	0.00333	0.435
***rrnS***	0.405	NA^2^	0.00078	NA^2^
***rrnL***	0.409	NA^2^	0.00163	NA^2^
**tRNA**	NA^1^	NA^2^	0.00060	NA^2^
**overall**	0.525	0.0490	0.00150	0.203

Five copepods, the PCGs of which have been entirely determined, were selected for interspecific analysis. For the PCGs, divergence of amino acid was compared for the interspecific analyses, and the nucleotide divergence was outlined for the intraspecific comparisons. Ratios of non-synonymous to synonymous substitutions for the 13 PCGs were compared. Genes were labelled as outlined in the abbreviations section. DB and ωB: p-distance and dN/dS among five copepods; DW and ωW: p-distance and dN/dS within *C. sinicus*.

^1 ^missing data due to the incomplete dataset;

^2 ^unfeasible parameter for the corresponding genes.

To examine the evolutionary forces acting on the mitochondrial PCGs of copepods, rates of non-synonymous substitution (dN) *versus *synonymous substitution (dS) were determined. The observed dN/dS ratios (Table 2) were consistently lower than one, increasing from 0.0026 for *cytb *to 0.0926 for *nad1*. This indicates a strong purifying selection within this lineage. Values of dN/dS for genes of sparse polymorphism (*cox1*-*3*, *cytb*) were generally lower, in agreement with the idea that highly divergent genes are normally subjected to less selective pressure.

### Ribosomal RNA genes

**Figure 3:**
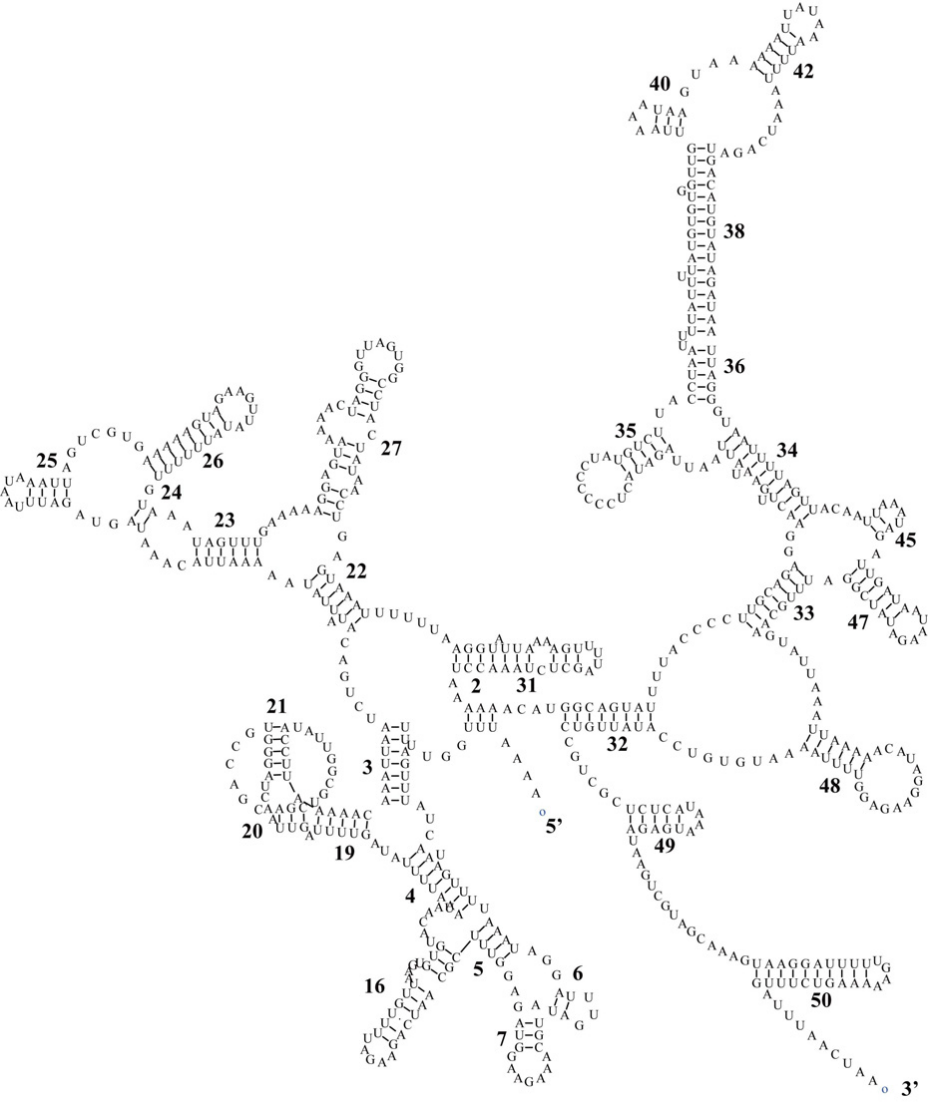
**The inferred secondary structure of the *rrnS *of *C. sinicus***. Inferred nucleotide bonds are illustrated by lines. The secondary structure was based on the model of Gutell (1994).

**Figure 4:**
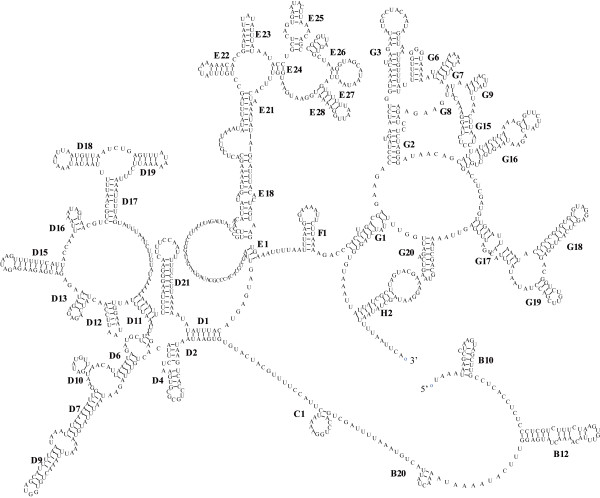
**The inferred secondary structure of the *rrnL *of *C. sinicus***. Inferred nucleotide bonds are illustrated by lines. Helix numbering follows that of De Rijk et al. (1997).

In the mitogenome of *C. sinicus*, the 16S ribosomal RNA (*rrnL*) and 12S ribosomal RNA (*rrnS*) genes are located between *trnV*/LNR1 and *trnG*/*nad6*, respectively. In arthropods, the rRNA genes are normally adjacent on the same strand, interleaved by a single *trnV*. However, the two genes are distantly separated on either strand in *C. sinicus*, which is rare in metazoans. Examples of the arrangement are mainly found in the primary lineages [28]. The size of *rrnS *and *rrnL *genes in *C. sinicus *were calculated to be 654 bp and 1,139 bp, respectively, on the basis of the alignment and comparison of their counterparts in *N. cristatus*. These lengths are similar to those of *P. nana*, but longer than corresponding lengths of other copepods. Consistent with PCGs, the two rRNA genes were determined to be highly divergent, with values of 0.405 and 0.409 for *rrnS *and *rrnL*, respectively (Table 2). The secondary structure of *rrnS *(Figure 3) was proposed on the basis of the model of Gutell [29], and *rrnL *(Figure 4) of the model of De Rijk et al. [30]. In accordance with their phylogenetic relationships, the secondary structures of *C. sinicus *rRNAs resembled those of crustacean [31] (*Daphnia pulex*) more closely than those of insect (*Drosophila yakuba*, secondary structures obtained from The European ribosomal RNA database [32]).

Compared with the insect *D. yakuba*, several compound helixes degenerate into a single one in the crustacean *rrnS *secondary structures. Both crustaceans lack helixes 8, 12, 39 and 41 whereas the counterparts are present in *D. yakuba*. All helixes present in *D. pulex *are shared by *C. sinicus *with the exception of helix 1. However, most loops and linking sequences between helixes are somewhat reduced, leading to a much shorter *rrnS *in *C. sinicus*. The alignment of *rrnS *genes for copepods indicates that 5' sequences upstream of helix 32 are more variable.

In terms of *rrnL*, upstream sequences of helix C1 were too ambitious to align. High diversity in this region has been reported in several species [33-35], where some or all helixes are truncated [33,35]. Helix G13, present in *D. yakuba*, is absent in *C. sinicus *and *D. pulex*. In addition, the compound Helix D13/D14 is replaced by one stem-loop, and Helix H3 is absent in *C. sinicus*. The greatest sequence conservation was at the 3' end from Helix G2 to H2, consistent with the idea that this region is the main component of the transferase centre [36].

### Transfer RNA genes

**Figure 5:**
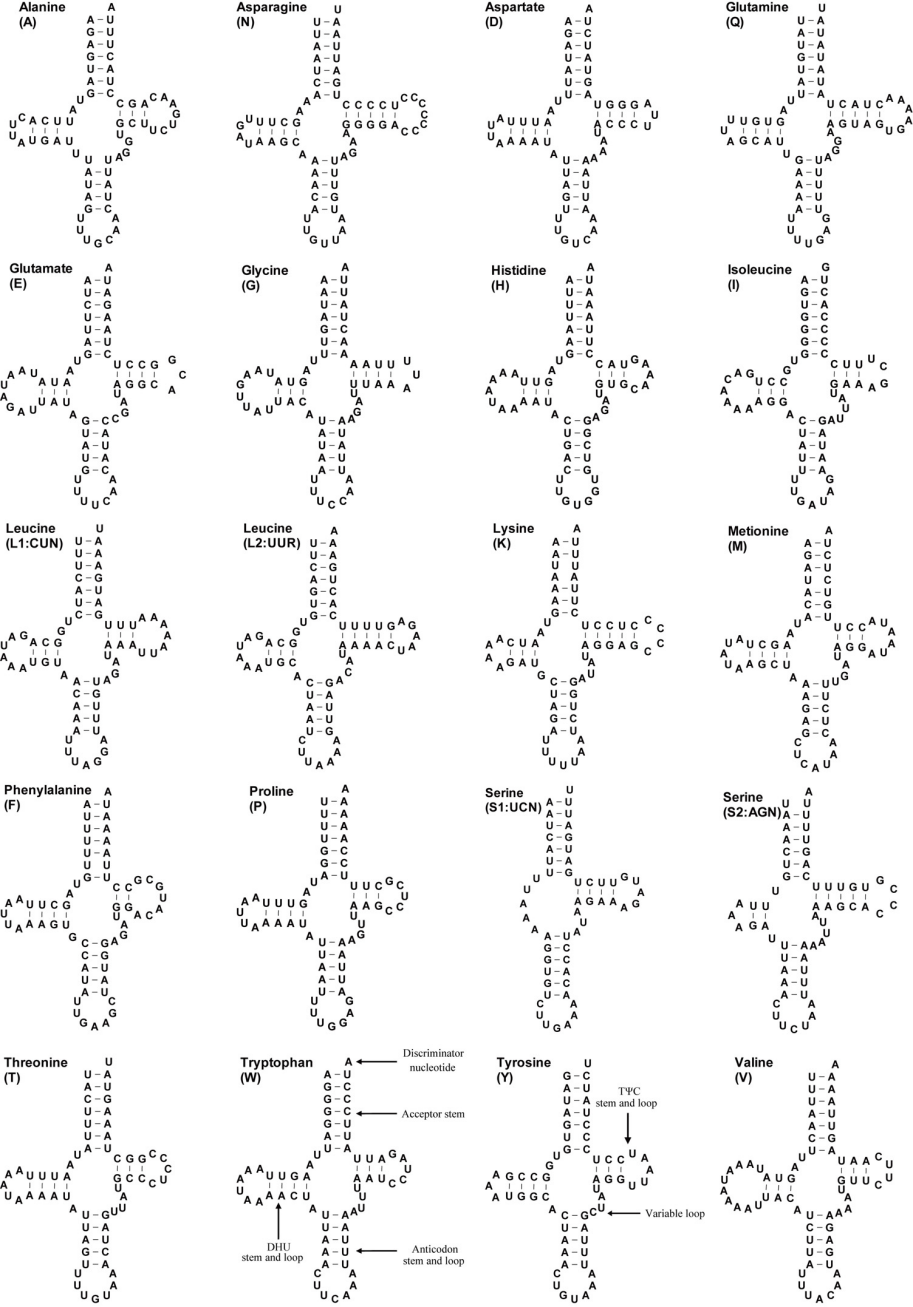
**Putative secondary structures of tRNAs in mitochondrial genome of *C. sinicus***. The tRNAs are labelled with the abbreviations of their corresponding amino acids. Each structural element is illustrated in *trnW *and *trnY*. Canonical cloverleaf structures are assumed in all tRNAs, with the exception of *trnS2 *(UCN).

Though only partially sequenced, 20 of the 22 mitochondrial tRNA genes have been identified in the *C. sinicus *mitogenome on the basis of their potential cloverleaf structures and anti-codons (Table 1, Figure 5). Four tRNA genes overlap with one to five shared nucleotides. The extreme example was identified at the junction between *trnY *and *trnE*. The overlapped portions can be repaired by a post-transcriptional editing process [36]. Gene lengths (57 to 68 nucleotides) and anti-codon usage are comparable with those generally observed in arthropods. However, *trnK *and *trnS1 *(AGN) utilize TTT and TCT rather than the more common CTT and GCT. Such substitutions on wobble positions can be found in other invertebrate mitogenomes [37]. As in other metazoans, anti-codons occasionally diverge from the most commonly used codons in degenerate codon families. For example, the most frequently used codon (AUA) for Met contradicts the corresponding anti-codon (AUG).

Complete cloverleaf structures containing the TΨC stem (mostly 3-5 bp) and loop (3-7 nt), variable loop, anti-codon stem (5 bp) and loop (7 nt), DHU (mostly 3-4 bp) stem and loop (highly variable from 3 to 10 nt), and the acceptor stem (7 bp), can be predicted for 19 tRNAs, whereas the DHU arm is absent in *trnS2 *(UCN). In addition, the DHU arm for another *trnS1 *(AGN) is highly reduced, leaving a short stem (2 nt) and a small loop (3 nt). Degenerative or unpaired DHU arms in *trnS *are considered to be a common condition in arthropods [38], and particularly in copepods [18,20]. As for other arthropods, the anti-codon is preceded by a uracil and followed by a purine in *C. sinicus*. Deviating from the canonical mitochondrial tRNAs with four nucleotides in the variable loops, 5 nt variable loops were identified in *trnI *and *trnS1 *(AGN), and 3 nt variable loops were identified in *trnS2 *(UCN).

### Non-coding sequences

Within the fragment determined, there were 6,270 bp non-coding sequences in total (approximate 30% of complete sequence) distributed among 23 intergenic regions. Six long non-coding regions larger than 100 bp were identified between *atp6 *and *rrnL *(LNR1; >2,959 bp; not sequenced completely), *cox1 *and *nad4L *(LNR2; 128 bp), *trnH *and *trnA *(LNR3; 1,770 bp), *nad3 *and *cox3 *(LNR4; 102 bp), *cox3 *and *nad4 *(LNR5; 762 bp), and *nad2 *and *atp8 *(LNR6; 172 bp). Six additional non-coding regions larger than 30 bp were discovered. The mitogenome of *C. sinicus *is one of the largest among arthropods owing to the prevalence and enlargement of non-coding regions. The concurrence of numerous large non-coding regions is unusual [39]. Because of the deletional bias, large inactive regions tend to be eliminated from mitogenomes so that they become economical [40]. Intergenic spacers are normally limited in number and size. As far as crustaceans are concerned, most mitochondrial genomes reported so far possess a single long non-coding region. Exceptions to this include *Speleonectes tulumensis*, *Hutchinsoniella macracantha *[41] and *Geothelphusa dehaani *[34]. The largest non-coding sequences, rather than CRs, are usually smaller than 40 bp [34]. To elucidate the origin of multiple non-coding regions, BLAST analysis was conducted on LNRs. With the exception of LNR2, in which a 26 bp stretch similar to other crustacean *rrnS *was screened, the BLAST analysis revealed that the LNRs of *C. sinicus *shared no significant similarities to any known sequences. Therefore, independent origins and evolutionary processes are likely to have given rise to the various non-coding regions.

Generally, large non-coding sequences act as control regions to initiate and/or regulate mitochondrial transcription and replication. However, functions of multiple heterologous non-coding regions are difficult to predict. AT-richness is broadly accepted as a characteristic for the identification of CRs. However, various cases have been reported [25], and appear to be common in copepods. Of five copepods, three possess equal (*L. salmonis *[20]) or lower (*T. japonicus *[18] and *T. californicus *[14]) A + T-contents in their control regions. Relatively lower AT-contents are present in *C. sinicus *LNRs with the exception of LNR2 (68.0%). Although conserved sequence blocks (CSBs) are common in control regions of metazoans [26,42], such conservative properties among copepods were not detected. Therefore, the control regions were screened on the basis of the secondary structure motifs.

Several secondary structure motifs commonly found in control regions of arthropods [42-45] were identified in LNR3 including: (1) a poly-T stretch 360 bp to the 3' end of LNR3; (2) a hairpin structure (Additional file 3) on the L-strand 140 bp downstream of the poly-T stretch; (3) conserved sequences at the lateral ends of the hairpin structure; and (4) a microsatellite locus following the hairpin structure with "AT" as the core repeat (14-28 repetitions). These motifs make LNR3 the most likely candidate for the mitochondrial control region. However, hairpin structures were identified in other LNRs, which could be related to the mode of regulation of replication and transcription. Considering the extreme complexity of the non-coding sequences in *C. sinicus*, more comparative and functional analyses are required to elucidate their exact roles during mitochondrial metabolism.

### Mitochondrial gene order

**Figure 6:**
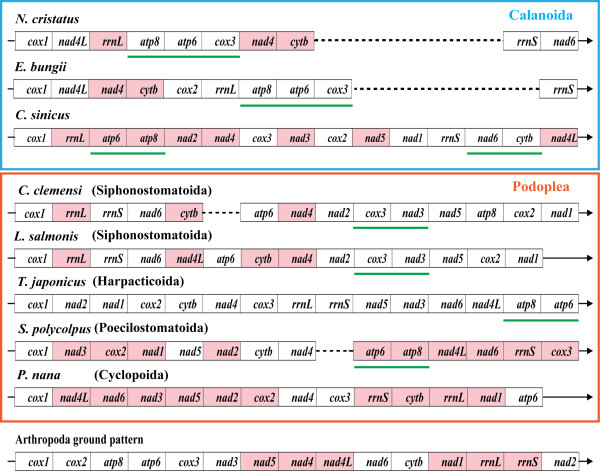
**Comparison of the mitochondrial gene arrangements in copepods and the putative arthropod ground pattern**. All tRNA genes and control regions were excluded for clarity. Gene segments are abbreviated as described in the main text, but are not drawn to scale. All elements were transcribed from left to right, with the exception of those depicted by shaded boxes. The horizontal lines illustrate gene blocks that are present in the Arthropod ground pattern. Large scale gene arrangements are apparent in copepods with all genes reshuffled.

In addition to the multiplication of LNRs, a notably shuffled gene order was present. Similar features were identified in a nonbilaterian species, *Nemateleotris magnifica *[46], but are rare in bilaterians [10]. Mitochondrial gene order rearrangements are common among crustaceans, particularly in copepods [19,21,23,25]. In the case of *C. sinicus*, the mitochondrial genome is heavily rearranged. Compared with other mitogenomes in the MITOME database (http://www.mitome.info), *C. sinicus *has a unique gene order. Comparison of the *C. sinicus *mitogenome to the ground pattern for arthropod mitogenomes [47] (Figure 6) revealed that the mitogenome was reshuffled. Translocations were identified for all tRNA genes in the mitogenome of *C. sinicus*. Among 36 gene boundaries, only two adjoining *atp6-atp8 *and *nad6*-*cytb *were retained. Moreover, 12 genes (34.2%) have developed a contrary transcriptional polarization: *trnD*, *atp6*, *atp8*, *nad3*, *trnA *and *nad2 *inverted from the L-strand to the H-strand, whereas *trnF*, *trnP*, *nad1*, *trnK*, *rrnS *and *trnQ *were shifted from the H-strand to the L-strand.

In accordance with the high rate of sequence divergence between lineages, scrambled gene orders were observed among copepods. Large-scale gene rearrangements were identified within the family Calanidae [23]. Owing to the small size and special secondary structures, tRNA genes are more mobile, and account for most translocations in crustaceans [25,34]. To avoid confusion initiated by reversal translocations of tRNA genes, the discussion of gene order is restricted to the protein-coding and rRNA genes (Figure 6). Complete reshuffling can be found in all copepod mitogenomes, leading to a divergent pattern of gene order in this group.

Translocations involving protein coding or rRNA genes are rare in metazoans [10]. Such dramatic rearrangement of genes in copepods challenges the view of conservation of mitochondrial gene order, as suggested by unusual gene translocations in molluscs [48,49]. When pairwise gene orders of copepods are compared, there are few common intervals (0 to 40) indicated by results from CREx [50]; exceptions are two siphonostomatoid species belonging to the family Caligidae (Additional file 4). No conserved synteny is shared by the copepod samples studied here, questioning their homologous status. The similarities of gene order within the family Calanidae and between the orders were compared, with no significant differences being identified. Therefore, the phylogenetic signal may be diluted by frequent gene rearrangements within the lineage. The lack of unambiguous synapomorphic gene arrangements in copepods precludes their use in phylogenetic analysis concerning Copepoda.

With regard to the rearrangement of mitochondrial genes, two major categories of mechanisms have been advanced: (1) tandem duplication followed by random or non-random deletion of excess genes [51,52]; and (2) non-homologous recombination [53,54]. The first scenario is improbable in view of the presence of inversion or the absence of a conserved synteny. Consequently, involvement of non-homologous recombination, which can invoke translocation and inversion, may be required. To date, there is no direct evidence of mitochondrial DNA recombination in copepods. However, new evidence supporting recombination is emerging in invertebrates including molluscs [55], nematodes [56] and arthropods [35]. Furthermore, the problematic *circular subgenomic fragment *identified in *C. sinicus *may provide additional insights concerning mitochondrial DNA recombination in copepods.

### Technical problems during laboratory work

During the experiments an 18.6 kb DNA sequence was amplified, with reverse complementarity in its 505 bp long lateral ends. Such a covalent sequence, which represents a circular DNA molecule, is normally considered as a marker for the achievement of mitochondrial sequencing. However, the 4.5 kb fragment at the 3' end of the sequence was incomplete, with several mitochondrial elements being absent (see Methods section for details). Extended sequences were successfully determined with step-out PCRs [57], and verified by Long-PCR. Therefore, the circular molecule was confirmed to be a sub-genomic fragment nested within the complete mtDNA (*circular subgenomic fragment*). The occurrence of the problematic *circular subgenomic fragment *could be explained by the following scenarios: First, the 4.5 kb fragment could be nuclear copies of mitochondrial fragments (*NUMT*s) [58]. *NUMT*s are normally composed of fragmented copies shorter than 4 kb [59,60]. Pseudogenes provide another notorious characteristic for the *NUMT*s [60]. However, sequences of the coding genes in this problematic fragment compare favourably with the counterparts obtained from the cDNA templates. Indel and nonsense mutations were not present. Therefore, because the sequences of coding genes in the 4.5 kb fragment are almost identical to those obtained from reverse transcriptase PCR (unpublished data), with only three substitutions in the 1372 nucleotides being compared, this first scenario is unlikely.

Second, the 4.5 kb fragment could be an artefact of PCR jumping when site-specific lesions exist or initial copies in the template are few [61]. Fresh materials were used for the amplification to reduce the possibility of PCR jumping as breaks in the template would give rise to the bouncing of primers. Unfortunately, abnormal nucleotides or stable stem-and-loop structures in the unidentified regions may have acted in a similar manner to breaks, causing the extending primer to jump to another template during PCR.

Finally, the *circular subgenomic fragment *could be the product of mitochondrial DNA recombination. Recombination is normally absent in mitochondrial genomes of metazoans, but convincing evidence for this process has emerged [56,58,62,63]. A defined feature of recombination is the breakage and rejoining of participating DNA strands [53]. According to Lunt's recombination model, subgenomic circular molecules with the same gene organizations but smaller in size could be generated during recombination. The erroneous fragment mentioned above is consistent with Lunt's model [53], suggesting that recombination occurred. The results highlight the possibility of mitochondrial DNA recombination in copepods.

Such puzzles may be common to all copepod studies and caution should be applied when using long PCR technology to define complete mitochondrial genomes. Additional long PCRs are required to confirm whether the mitochondrial genome sequence is complete.

### Phylogenetic analysis

**Figure 7:**
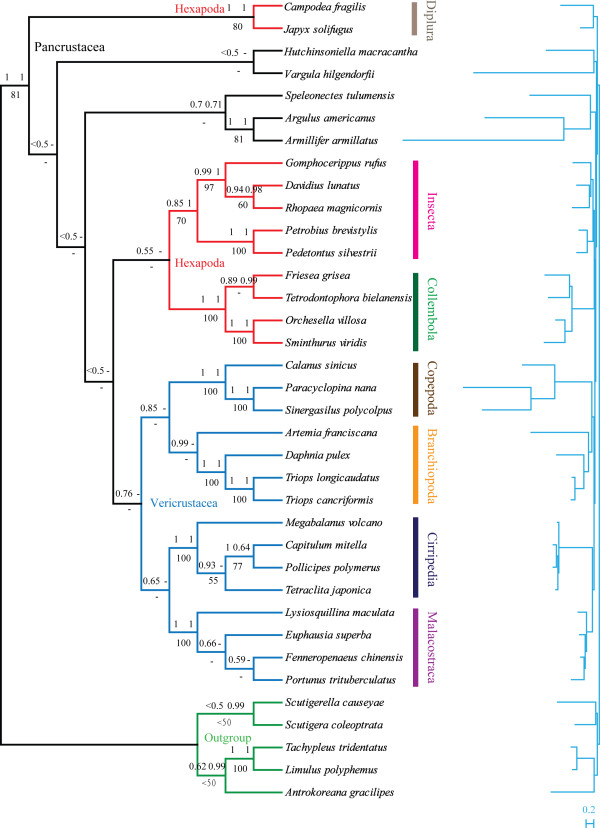
**Phylogenetic relationships of major pancrustacean lineages inferred from concatenated amino acids of 12 mitochondrial protein coding genes (original dataset)**. Two Chelicerate and three Myriapoda species were used as out-groups. The topology was represented by the result obtained from PhyloBayes under the model of CAT + mtArt. Each group of three numbers at the branch nodes (clockwise) refer to the Bayesian posterior probabilities using PhyloBayes and MrBayes, and bootstrap support values using PHYML. The scale bar represents substitutions per site. '-' indicates the node was not supported by the corresponding analysis.

**Figure 8:**
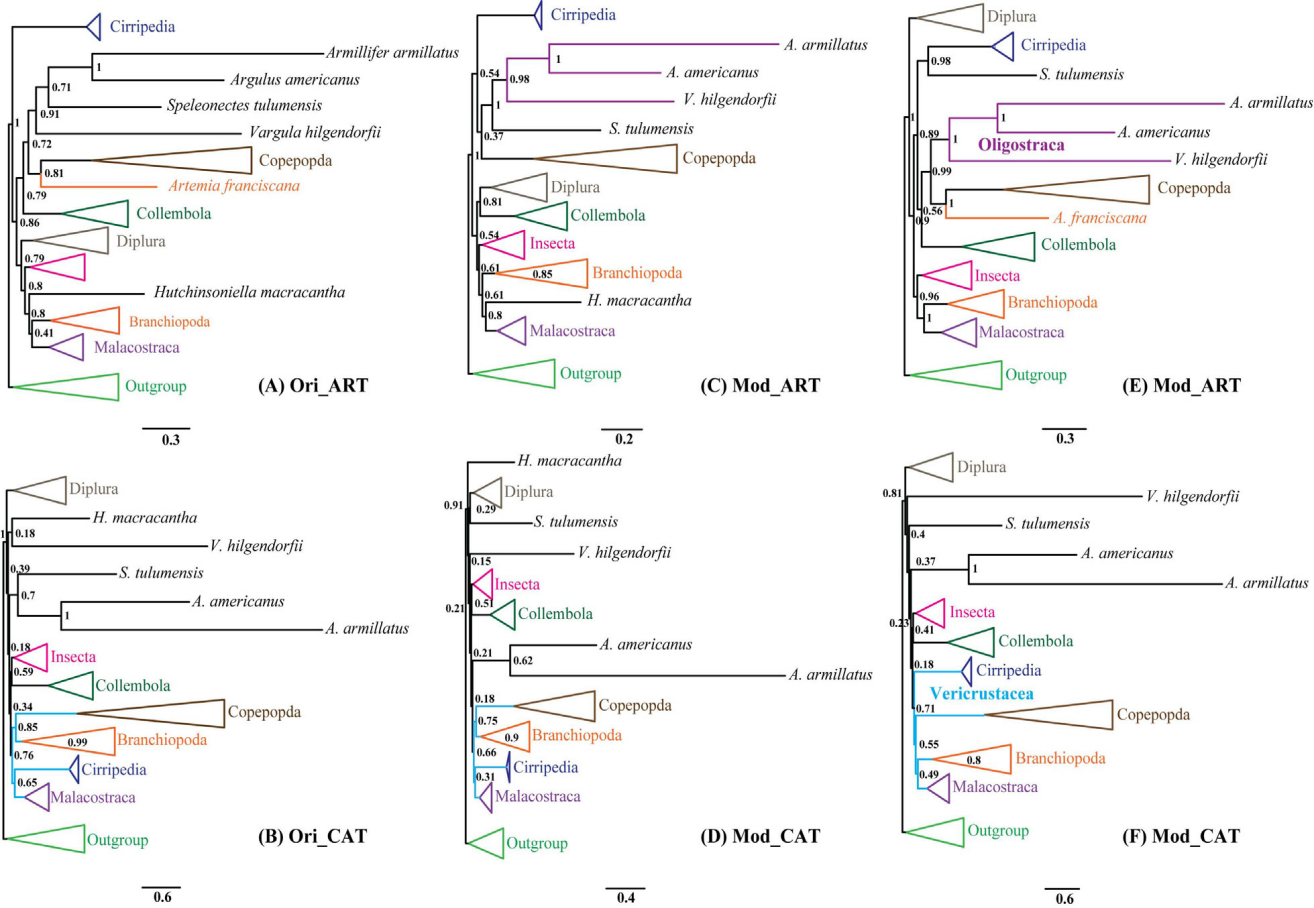
**The position of Copepoda and phylogenetic relationship of pancrustacean lineages are sensitive to the methods selected**. Analyses were performed on three datasets: (1) with complete amino acid alignments (Ori); (2) with only sites carrying a moderate evolutionary rate (Mod); (3) with strand-biased proteins excluded (Bal) under the mtArt (ART) or the CAT + mtArt (CAT) models. (A) Ori dataset under the ART model; (B) Ori dataset under the CAT model; (C) Mod dataset under the ART model; (D) Mod dataset under the CAT model; (E) Bal dataset under the ART model and (F) Bal dataset under the CAT model. A schematic version of the Bayesian trees is presented with a number of well-supported lineages collapsed for clarity.

Homogeneity of the stationary frequencies across the tree is a baseline for current phylogenetic models. Therefore, amino acid alignments were used for inference of phylogeny as they are more homogenous among different lineages than nucleotide content [64]. As presented in Figures 7 and 8, monophyly of Pancrustacea and most of its high-level subtaxa including the classes Collembola, Diplura, Insecta and Malacostraca, and the subclasses Copepoda and Cirripedia, were supported irrespective of the model and method applied. Bootstrap support values (BP) from maximum likelihood (ML) analyses were usually lower in the current analysis, suggesting that the phylogenetic signal was weak or that a competing artificial signal was present. ML analyses are believed to be vulnerable to several factors including lineage-specific evolutionary rate heterogeneities and nucleotide compositional heterogeneities, which can impede the recovery of phylogenetic signals [16,64]. Hence, this study concentrated on the topology recovered by Bayesian inference (BI), but included ML trees.

**Figure 9:**
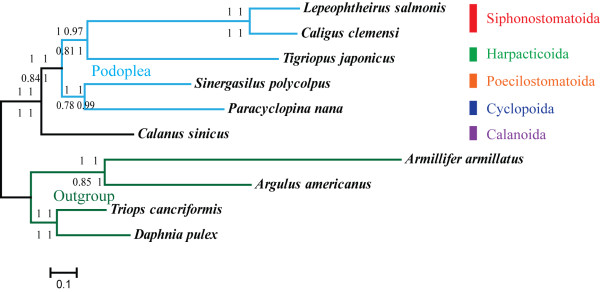
**Phylogenetic relationship of five component orders belonging to the subclass Copepoda based on Bayesian analysis**. Two Branchiopoda and two Maxillopoda species were used as out-groups. Trees from various analyses are of the same topology. Only the tree inferred from original dataset using the model of CAT + mtArt is shown. Each group of four numbers at the branch nodes (clockwise) represents the Bayesian posterior probabilities of four analyses, Ori_ART, Ori_CAT, Bal_ART and Bal_CAT. The names were abbreviated as depicted in Figure 8.

Monophyly of copepods was well resolved in the results and in a former mitochondrial phylogenetic inference with expanded out-group sampling (Additional file 5). Consensus has been reached for the monophyly of Copepoda on the basis of morphological and molecular evidence [3,4,65], whereas the phylogenetic relationships among component orders are still controversial. As far as the phylogenetic relationships among copepods are concerned, congruent results were obtained from different analyses during the current research (Figure 9). Harpacticoida (*T. japonicus *) and monophyletic Siphonostomatoida ( *L. salmonis *and *C. clemensi*) grouped together, with the cluster containing Poecilostomatoida ( *S. polycolpus*) and Cyclopoida ( *P. nana *) as their sister clade. The grouping of the four orders, excluding Calanoida, confirms the monophyly of Podoplea, which is characteristically tagged by the podoplean tagmosis [3]. The basal splitting of Copepoda separated Calanoida from Podoplea, reflecting the primary status of Calanoida within copepods. With regard to inter-ordinal phylogenetic relationships within Podoplea, Huys et al. proposed an early split between MCG-Clade (Misophrioida, Cyclopoida and Gelyelloida) and MHPSM-Clade (Mormonilloida, Harpacticoida, Poecilostomatoida, Siphonostomatoida and Monstrilloida), where Poecilostomatoida and Cyclopoida separated into distinct lineages soon after Podoplea was formed [3,4,6,65]. However, similar to the results from Huys, based on 18S rRNA [66], Cyclopoida and Poecilostomatoida exhibited closer affinity in this study, supporting Boxshall's hypothesis to reunite Poecilostomatoida into Cyclopoida. This view is gaining support from several independent analyses [7,67]. Accordingly, the present results support the hypotheses for (outgroups, Calanoida, ((Cyclopoida, Poecilostomatoida), (Harpacticoida, Siphonostomatoida)).

The position of Copepoda within Pancrustacea is still unclear; the present analyses produced conflicting results using different methods. The uncertainty regarding the position of Copepoda within Pancrustacea is in part due to heterogeneity in evolutionary rates and nucleotide compositions within the lineage [64], and may be exacerbated by the derived nature of copepod mitochondrial sequences. Considering that copepods possess notably biased nucleotide compositional profiles, the recovery of the phylogenetic signal may be impeded by lineage-specific compositional heterogeneity. However, exclusion of strand-biased amino acids does not change the relative position of Copepoda (Figure 8), indicating that heterogeneous nucleotide composition may not play a key role in misleading phylogenetic analysis. Nevertheless, accelerated substation rates of copepods do mask and erode phylogenetic signals by attracting long-branched taxa together. One example concerns the previously well-accepted monophyletic group Branchiopoda [27,65], which was resolved polyphyletically by clustering *A. franciscana *with copepods in the original and balanced dataset under the mtArt model. However, analyses performed with only characters carrying a moderate evolutionary rate or with the CAT + mtArt, which has been confirmed as an effective model to overcome the effects of Long Branch Attraction (LBA) [68], consistently resolved a monophyletic Branchiopoda clade. Consequently, a possible LBA artefact could be introduced by the accelerated rate of evolution of the mitochondrial genomes of the sampled copepods and branchiopod. Therefore, the clustering of *A. franciscana *and copepods was regarded as a phylogenetic artefact due to the LBA rather than a sister grouping.

As for the position of Copepoda, four possible sister groups have been proposed in the present study (Figure 8): (1) Oligostraca including Ostracoda, Pentastomida, Branchiura [69], (2) Oligostraca plus Remipedia under the model of mtArt and (3) Branchiopoda, (4) Branchiopoda plus Malacostraca under the model of CAT + mtArt. It should be noted that by inspecting branch lengths, Copepoda, Oligostraca and Remipedia are rapidly evolving lineages. A decrease in support (PP = 0.37) for the close affinity of Oligostraca and Copepoda was observed in mtArt trees with the moderate-rate sites. Therefore, their grouping may be misleading owing to the artefact concerning the LBA, as the mtArt model is vulnerable to LBA artefacts. Consequently, although only moderately supported (PP from 0.66 to 0.76), the results obtained with the CAT + mtArt model, in which Copepoda was clustered in the monophyletic clade Vericrustacea, which joins Malacostraca, Branchiopoda, Thecostraca and Copepoda, are accepted in the present study. These results are compatible with a previous phylogenetic analysis based on nuclear protein-coding sequences [69].

The relationships among pancrustacean lineages are highly unstable, but several interesting findings resolved using various methods are noted. A monophyletic origin of Pancrustacea (Figures 7 and 8) is strongly supported by the current analyses, as recovered by a number of molecular studies on the bases of sequence data [16,65,70] or mitochondrial gene orders [71]. In accordance with other mitochondrial studies [72], monophyly of Hexapoda and Crustacea was rejected in the present study, although relationships among the component lineages remain unstable. However, it is noteworthy that the affinity of Insecta and Collembola was resolved under the model of CAT + mtArt, while the grouping of Collembola with Diplura was recovered under the model of mtArt using only moderate-rate sites. These results prevent the rejection of the monophyly of Hexapoda on the basis of mitochondrial genomic data alone. Contradictory conclusions from mitochondrial sequences and nuclear sequences [69,70,73] mean that the monophyly of Hexapoda and Crustacea are open questions. Traditional but controversial Maxillopoda was resolved paraphyletic/polyphyletic in the present analyses, where it can be sub-divided into three groups including Copepoda, Cirripedia and Pentastomida plus Branchiura. This division of Maxillopoda is in agreement with recent studies based on combined data of 18S rRNA and two mitochondrial markers [65], and nuclear protein-coding genes [69].

### Intraspecific sequence variability and its utility for population genetic analysis

**Figure 10:**
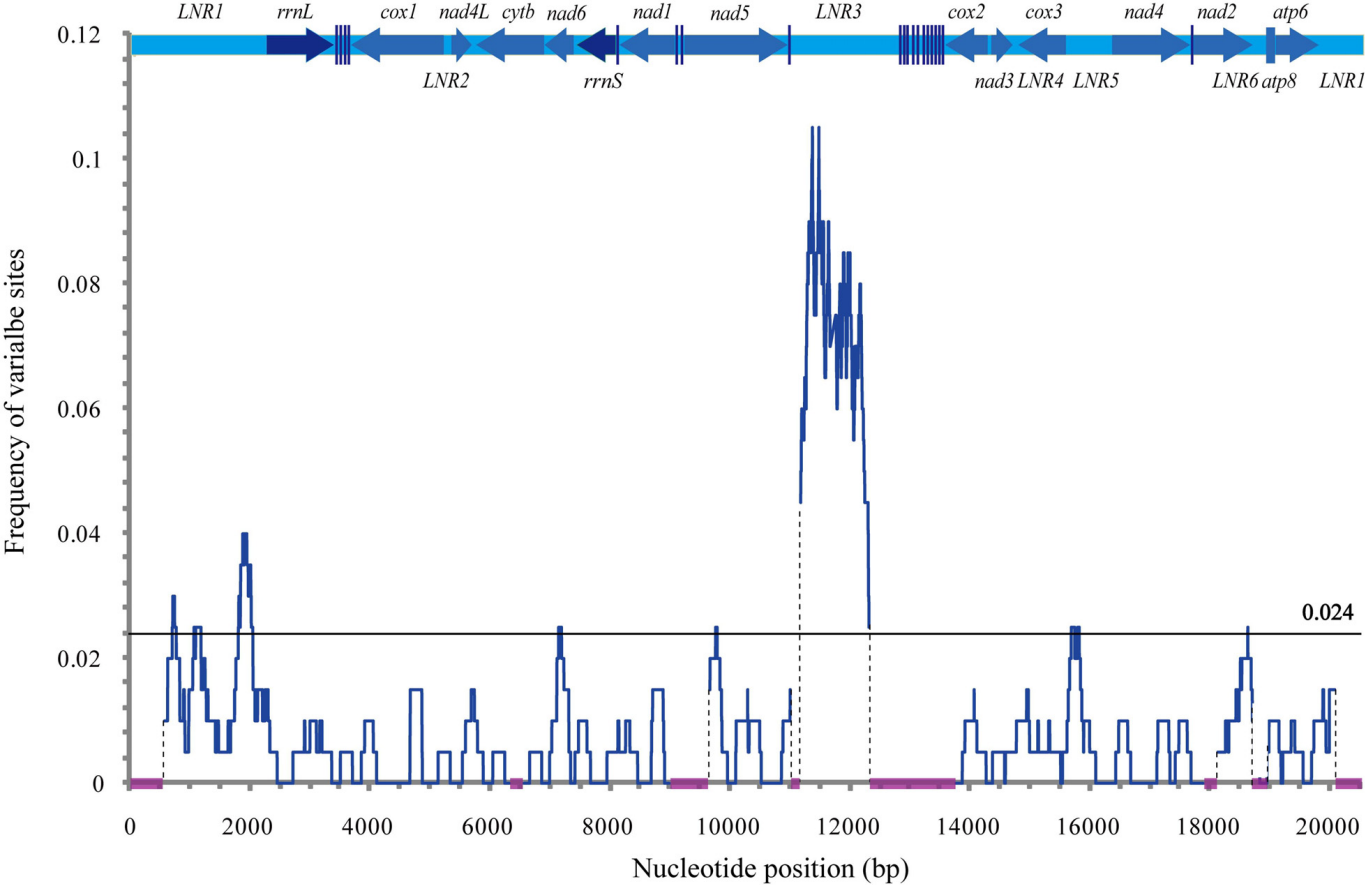
**Plot representation of the frequency of variable sites across the mitochondrial genome of *C. sinicus***. The sliding-window was 200 bp in length and slid 2 bp at a time. Eleven individuals from four populations were used to determine the intraspecific distribution pattern of the variable sites. The bar at the top illustrates the position and orientation of each gene. The shaded boxes on the x-axis indicate sections that were not covered during the comparison. The horizontal line indicates the mean value of the frequency.

The major loci were scanned on the basis of the alignment of 11 mitogenomes from four populations for proper molecular markers. No evidence for recombination in the mitogenome of *C. sinicus *was detected. Within the 16,670 bp alignment [GenBank: HQ619228-HQ619237] there were a total of 397 variable sites including 295 single nucleotide polymorphisms (SNPs) comprising 191 nucleotide substitutions and 104 insertion/deletion polymorphic sites, in addition to three microsatellite motifs. A sliding-window analysis was performed to map the distribution of variable sites among 11 individuals in DNAsp [74] (Figure 10). The mean frequency of the variable sites was relatively low (approximately 0.024). LNR3, harbouring two microsatellite motifs, was most variable, while *nad4L *was the most conserved with no changed sites. The "hotspots" bearing the highest frequency of variable sites were bases 11216-12260 with 226 variable sites in 1045 bases (1 in 4.6), 1848-2235 with 22 variable sites in 388 bases (1 in 17.6), and 649-862 with 10 variable sites in 214 bases (1 in 21.4). The former corresponds to LNR3, and the others span LNR1 and upstream of *rrnL*. The gene encoding *cox1 *is used wildly as molecular marker for analysis at the population level [13]. However, a 752 bp stretch spanning 4016-4767, which is located in the gene of *cox1*, has no variable site. The results revealed the conserved nature of *cox1 *and eliminated the possibility of its utility in population genetics for *C. sinicus*. Constant phylogenetic signals, which distinguish haplotype groups, were detected in the hyper-variable regions (unpublished data), underpinning their utility for population analysis.

Non-coding regions are most variable, followed by PCGs and rRNA genes, with tRNA genes being the most conserved (Table 2). No deletions or insertions were identified in the coding regions. Of the 191 nucleotide substitutions, 50.2% were identified as parsimony informative. A distinct bias for nucleotide transitions over transversions was evident, with 87% substitutions being transitions.

Of the 55 SNPs in PCGs, only 18 can give rise to amino acid substitutions. The majority of the SNPs (62.1%) are located in wobble codon positions. Only 14.0% were identified in second positions, all of which lead to amino acid substitutions. Among PCGs, *nad6 *is most divergent (0.00333). As presented in Table 2, intraspecific dN/dS ratios were either zero or at relatively high levels, ranging from 0.157 for *atp6 *to 0.914 for *nad4*. The overall intraspecific dN/dS ratio was 4.14 times larger than the counterpart between species, which is compatible with a nearly neutral model in which most amino acid substitutions are slightly deleterious [75].

In terms of the 11 nucleotide substitutions in structural RNA genes, seven were situated in stems and the remainder in loops. Three substitutions in stems induced deterioration of stem connections. However, they were all present at lateral margins, indicating little influence on the overall secondary structures. As mentioned above, such mis-pairing is considered to be restored during transcription.

In addition to substitution and indel variations, three microsatellite motifs were identified at regions from bases 11492 to 11504, 11645 to 11756 and 13066 to 13095. "TCC" unit is the core repeat for the first motif, while "TA" acts as a core repeat for the others. The second microsatellite is most variable with repeat numbers from 10 to 56. With additional subtle sequence variations within the motif, the 11 sequenced microsatellite loci can be sub-divided into seven alleles.

## Conclusions

This study presents the first nearly complete mitochondrial genome of a Calanoida species. The *circular subgenomic fragment *obtained invoked caution when analyzing the mitogenome of copepods using long PCR technology, and may offer additional evidence for mitochondrial recombination.

Although the contents and lengths of individual genes are similar to other arthropods, the mitogenome of *C. sinicus*, one of the largest mtDNAs in crustaceans, is enlarged by the prevalence and extended length of non-coding regions. The concurrence of multiple non-coding regions and reshuffled gene arrangement results in the mitochondrial genome of *C. sinicus *being remarkably distinctive from other arthropods. Mitochondrial DNA recombination may have played an important role in shaping the present mitochondrial profile of *C. sinicus*. The lack of synapomorphic gene arrangements among copepods raises questions concerning the use of gene order as a useful molecular marker for deep phylogenetic analysis.

Recovery of the phylogenetic signal in mitochondrial genomes may be affected a variety of reconstruction artefacts including lineage-specific heterogeneities for nucleotide composition and evolutionary rate. In particular, the LBA artefact influenced the results during analysis. Several methods were designed to reduce the dilution effect of the reconstruction artefacts. Although unstable, some inspiring congruent results were noticed in the analyses. Monophyly of copepods and the basal split between Calanoida and Podoplea were successfully resolved. The close affinity between Cyclopoida and Poecilostomatoida in the present study supports Boxshall in reassigning the latter subordinate to the former. Copepoda was clustered within the monophyletic clade Vericrustacea, although relationships among the lineages remain ambiguous. Falsification of Maxillopoda was confirmed by unveiling its paraphyletic/polyphyletic nature. However, owing to the limited phylogenetic signals in the mitochondrial data sets, no consensus concerning relationships among pancrustacean lineages was reached.

Within the 16,670 bp alignment there are a total of 397 variable sites. Indel variations are present in non-coding regions and transitions dominate the nucleotide substitutions. Three "hot-spots", particularly the hyper-variable microsatellite locus in LNRs, provide rich polymorphisms for population studies.

## Methods

### Sample Collection and DNA Extraction

*C. sinicus *for mitogenome characterization were collected from the Yellow Sea (35.9N, 122.9E) with a 500 μm mesh zooplankton net during a summer cruise in 2006. The samples were preserved at -80°C pending DNA extraction. To compare the intraspecific polymorphism pattern of different loci among populations, *C. sinicus *from Yangtze estuary (28.6N, 122.1E; 4 individuals), North Yellow Sea (38.7N, 120.7E; 3 individuals) and Korea (36.9N, 126.3E; 3 individuals) were selected.

Fifty individuals of *C. sinicus *from the same population (the Yellow Sea) were pooled to prepare a DNA template for mitogenome sequencing. To avoid the potential influence of nuclear DNA sequences on mitochondrial origin, crude mitochondria were roughly separated from cell debris and nuclei using differential centrifugation with a commercial tissue mitochondria isolation kit (Beyotime, C3606). mtDNA was extracted using the DIAamp DNA Micro Kit (Qiagen, Valencia, CA) following the manufacturer's instructions. For intraspecific comparison, genome DNA was extracted individually.

### PCR amplification and sequencing

Partial sequences of the genes *atp6*, *cytb, nad4 *and *rrnS *were determined using the primers presented in additional file 6. On the basis of the sequence data obtained, long PCR primers (Additional file 6) were designed to amplify the entire *C. sinicus *mitogenome. Two PCR fragments with lengths of 3.8 kb and 11 kb were successfully amplified with the combination of primer cs_*cytb*F1 plus cs_16sR1 and primer *cytb*f3 plus cs_*nad4*f. PCR reactions were performed using a Mastercycler Pro gradient machine (Eppendorf) in a 50 μl system containing 30 pmol of each primer, 3 nmol of each dNTP, 1.5 units of LA *taq *polymerase and approximately 20 ng of mtDNA template in 1X La taq buffer supplied by Takara. The cycle profile was initiated with a denaturation step of 94°C for 3 min, followed by 33 cycles of 95°C for 20 s, 58°C for 30 s, 68°C for 1 min/1 kb, and terminated with a final extension cycle of 72°C for 10 min. The product was purified with an E.Z.N.A. gel extraction kit (Omega) and sequenced directly by the primer walking approach (Additional file 6).

Additional primers facing outward were designed from both ends of the contig. A 4.5 kb fragment, with which all contigs could be cyclized, was amplified. However, the absence of *atp6 *in the amplicon made the results unreliable. Illegality of the fragment was confirmed by failure of amplification with primers from dubious regions.

Step-out PCR techniques [57] were applied for the remaining mitogenome in two directions, with the primers targeting lateral margins. Despite repeated attempts, the amplification terminated in certain regions on both sides. PCR products smaller than 5 kb were sequenced directly. Some short PCR fragments were also cloned using PMD-18T (Takara) vector before sequencing when they were unable to be sequenced directly. The 11 kb PCR product was sheared into small fragments of 1-3 kb using HydroShear (Genomic Solutions), and then cloned with PUC19 vector (Fermentas) after being end-repaired with T4 DNA polymerase (NEB) following the manufacturer's protocol. Forty clones were sequenced with an ABI 3730 sequencer from Biosune (Shanghai) company.

On the basis of the mitogenome sequences obtained, four primer combinations from relatively conserved regions were designed for screening polymorphism loci in the *C. sinicus *mitogenome. Fragments of 1.1 kb to 9.5 kb in length from 10 individuals were sequenced using the methods described above. Base calling was performed with phred, and the reads were assembled in phrap with default parameters [76,77]. All assembled sequences were manually verified with the aid of CONSED to remove misassembles [78]. The nearly complete mitochondrial genome of *C. sinicus *has been deposited in GenBank with the accession number [GenBank: GU355641].

### Sequence analysis and annotation

DNA sequences were analyzed using the software package Lasergene ver. 7.1.0 (DNASTAR, Inc. Madison) and Vector NTI Advance 9 (Invitrogen, Carlsbad, CA). Locations of protein coding genes were preliminarily identified by the ORFs finding method from GeneQuest, followed by BLAST searching on GenBank datasets. The locations were refined using multiple alignments to other crustacean nucleotide sequences. tRNA genes were identified by their proposed cloverleaf secondary structure and anti-codon sequences using tRNAscan-SE1.21[79] and ARWEN [80] with relaxed settings, and confirmed manually. Two rRNA genes were determined by comparing with other annotated crustacean mitogenomes, and reconfirmed using their secondary structures. Inferred rRNA sequences were aligned with other crustaceans, whose secondary structures have been launched (*rrnS *and *rrnL *obtained from the rRNA database: http://www.psb.ugent.be/rRNA/index.html) by means of the program DCSE [81]. The program RnaViz 2 [82] was used to draw secondary structures of tRNAs and rRNAs. Secondary structures of the putative control region, according to the model for arthropods [25], were estimated using UNAfold [83]. Gene divergence and synonymous and non-synonymous substitution rates in the protein coding genes were calculated with DnaSP 4.0 and PAML 4.3 [84].

### Sequence alignment and phylogenetic analysis

In addition to the complete mitochondrial genome of *C. sinicus *presented here, mitogenome sequences of another 105 arthropods were retrieved from GenBank. Information concerning phylogenetic position, gene order, nucleotides and amino acids of individual genes, and sizes of mitogenomes, was extracted from the combined datasets using purpose-built perl scripts (Additional file 7). To avoid artefacts due to asymmetric nucleotide composition, the nucleotide content of a concatenated sequence of PCGs from the initial dataset were compared. Principal components analysis (PCA) was performed with contents of component nucleotides as variables (Additional file 8). The results were used as a guide for sampling taxa with relatively homologous nucleotide compositions. The nucleotide composition and strand asymmetry of some maxillopods are not as balanced, but they were included for complete taxon coverage. A sample containing 36 species including three copepods was selected. Amino acid sequences of individual proteins were aligned using Probalign under the default settings for protein [85]. *atp8 *was not included as it was absent from some taxa sampled. A dataset (Original dataset) of 2646 amino acids with posterior probabilities above five was accepted for the subsequent phylogenetic analysis. To explore the signal in the dataset and clarify the placement of Copepoda, two additional datasets were introduced. For the first dataset (Balanced dataset), only proteins whose nucleotide composition was not significantly strand-biased were included. Since too-rapidly and too-slowly evolving sites may affect phylogenetic analysis [64], another dataset (moderate-rate dataset) was constructed by removing classes of rapidly and slowly evolving sites using the slow-fast approach [64,86], in which sites were partitioned into quartiles and only those from the two internal ones were accepted.

To understand phylogenetic relationships among copepods, two smaller datasets were built (Original and Balanced datasets). These datasets consisted of six copepods whose complete mitochondrial genomes have been (almost) entirely determined, in addition to two branchiopods and two maxillopods as out-groups. For the intraspecific sequence variability analysis, reads from another 10 individuals were assembled and manually aligned in BioEdit (North Carolina State University, NC) using the *C. sinicus *mitogenome as a template. Alignment was performed on individual genes with sequences from other copepods using Probalign to estimate sequence divergence of various loci.

According to preliminary analysis, the CAT + MtArt model and MtArt model fit the data best and were selected for further analysis. Bayesian analyses were carried out using MrBayes (MtArt model) and PhyloBayes (CAT + MtArt model), with an among-site rate variation under a gamma distribution using four activated categories. Two independent MCMC chains were run simultaneously to determine whether the searching reached stabilization, and were stopped when all chains converged (maxdiff less than 0.2, but in most of the cases less than 0.1 for PhyloBayes; standard deviation [SD] of split frequencies lower than 0.01 for MrBayes). If not, runs were continued until more than 5000 sample points were available per run. The ML analysis was carried out with PHYML 3.0 with 200 bootstrap replicates.

## Abbreviations

*atp6 *and *8*: ATPase subunit 6 and 8; bp: base pair (s); BI: Bayesian inference; BP: Bootstrap; *cox1*-*3*: cytochrome c oxidase subunits I-III; *cytb*: cytochrome b; LBA: long-branch attraction; *rrnL*: 16S ribosomal RNA; LNR: large non-coding region; ML: maximum likelihood; mitogenome: mitochondrial genome; mtDNA: mitochondrial DNA; nt: nucleotide (s); *nad1*-*6 *and 4L: NADH dehydrogenase subunits 1-6 and 4L; ORF: open reading frame; PCG: protein coding gene; PCR: polymerase chain reaction; PP: Bayesian posterior probabilities; rRNA: ribosomal RNA; SNP: single nucleotide polymorphism; *rrnS*: 12S ribosomal RNA; tRNA: transfer RNA; *trnX *(where X is replaced by single letter amino acid code of the corresponding amino acid): tRNA gene.

## Authors' contributions

MXW and SS contributed to the conception and design of the study. MXW and XS conducted the majority of the laboratory work and are responsible for the data analysis. SS and CLL supervised the study and provided technical support during experiments. SS, CLL and MXW cooperated with the writing of the manuscript. XS provided important advice on revision of the manuscript. All authors read and approved the final manuscript.

## Supplementary Material

Additional file 1 **Comparison of the length, A + T-content and nucleotide compositional bias of mitochondrial genomes among copepods**. Values were obtained from the corresponding GenBank files. Detailed values are present for the complete genomes, overall PCGs, separate codon of PCGs and structural rRNA genes. ^NA ^missing data due to incomplete sequencing of the mitogenome. Click here for file

Additional file 2 **Codon usage for the protein-coding genes in the mitogenome of *C. sinicus***. n indicates the total number of codons used in all 13 mitochondrial protein-coding genes. Click here for file

Additional file 3 **Stem-loop structures in the putative control region of *C. sinicus***. Potential hairpin structures within the LNR3 between *trnH *and *trnA *were constructed using UNAfold. Conserved motifs in 3' and 5' flanking sequences are underlined. The depicted region corresponds to the complementary strand of 11522 -11708 bp in the submitted sequence. Click here for file

Additional file 4 **Pairwise comparison of mitochondrial gene orders among copepods**. tRNA genes were not included. Common intervals (above), defined as the number of shared gene blocks inside a block independent of their gene orders, were calculated for comparison. Click here for file

Additional file 5 **Phylogenetic tree presenting the monophyly of Copepoda and its position within arthropods inferred from concatenated amino acids of 12 mitochondrial protein coding genes**. Tree topologies produced by ML (mtArt model under PHYML) and BI (mtArt model under MrBayes) were compactable. Numbers at the branch nodes refer to Bayesian posterior probabilities and bootstrap support values from left to right. Click here for file

Additional file 6 **List of primers used to determine the mitogenome of *C. sinicus***. Numbers refer to the nucleotide positions of 5' end of primers. Click here for file

Additional file 7 **Perl scripts to extract mitochondrial genomic information from GenBank files**. Bioperl modules are required. Click here for file

Additional file 8 **Nucleotide compositional properties of the candidates for phylogenetic analysis illustrated by principal components analysis (PCA)**. PCA ordination was based on the proportion of separate nucleotides. PC1 (principal component 1) explained 87% of the total variations and PC2 explained 10% of the total variations. Species were sampled predominantly inside the red ellipse. Click here for file
