# Supplementary figures and images for: Distinctive mitochondrial genome of Calanoid copepod Calanus sinicus with multiple large non-coding regions and reshuffled gene order: Useful molecular markers for phylogenetic and population studies
Source: BMC Genomics. 2011 Jan 27;12:73. doi: 10.1186/1471-2164-12-73 (PMC3041745; doi:10.1186/1471-2164-12-73)

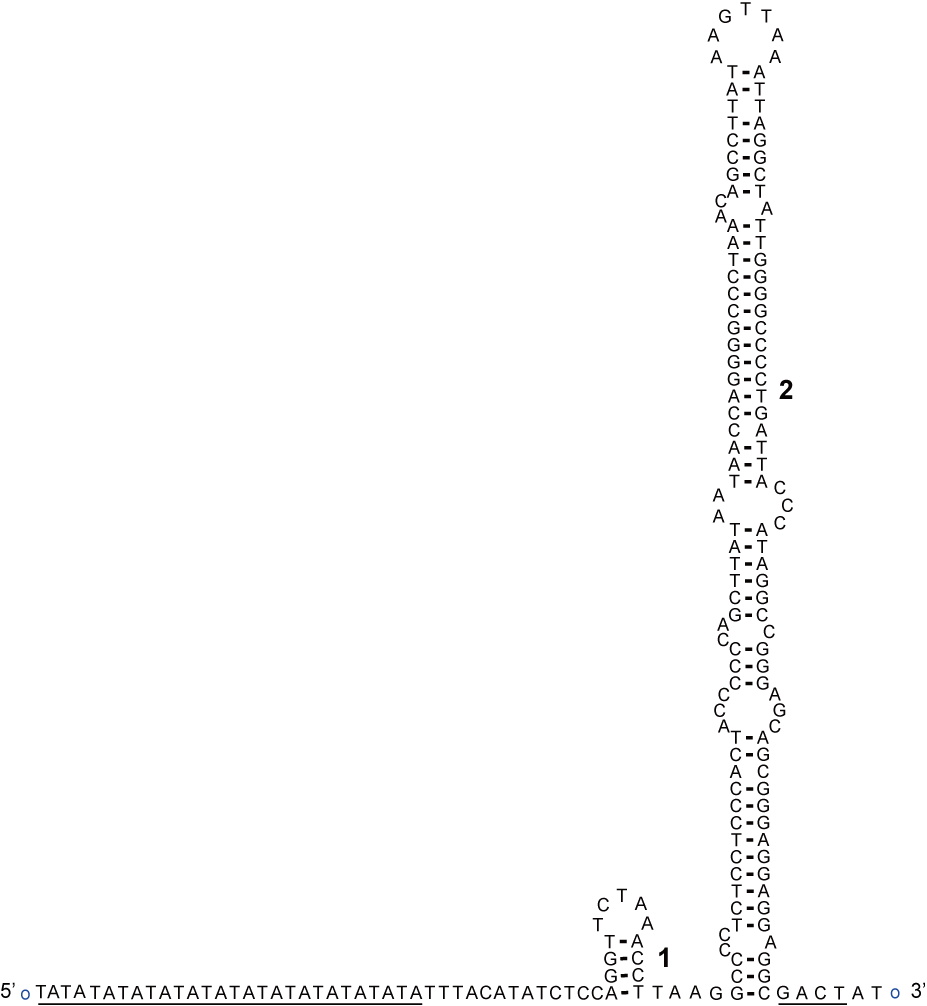

Supplement: Additional file 3 — Stem-loop structures in the putative control region of C. sinicus. Potential hairpin structures within the LNR3 between trnH and trnA were constructed using UNAfold. Conserved motifs in 3' and 5' flanking sequences are underlined. The depicted region corresponds to the complementary strand of 11522 -11708 bp in the submitted sequence. [file 1471-2164-12-73-S3.PNG]

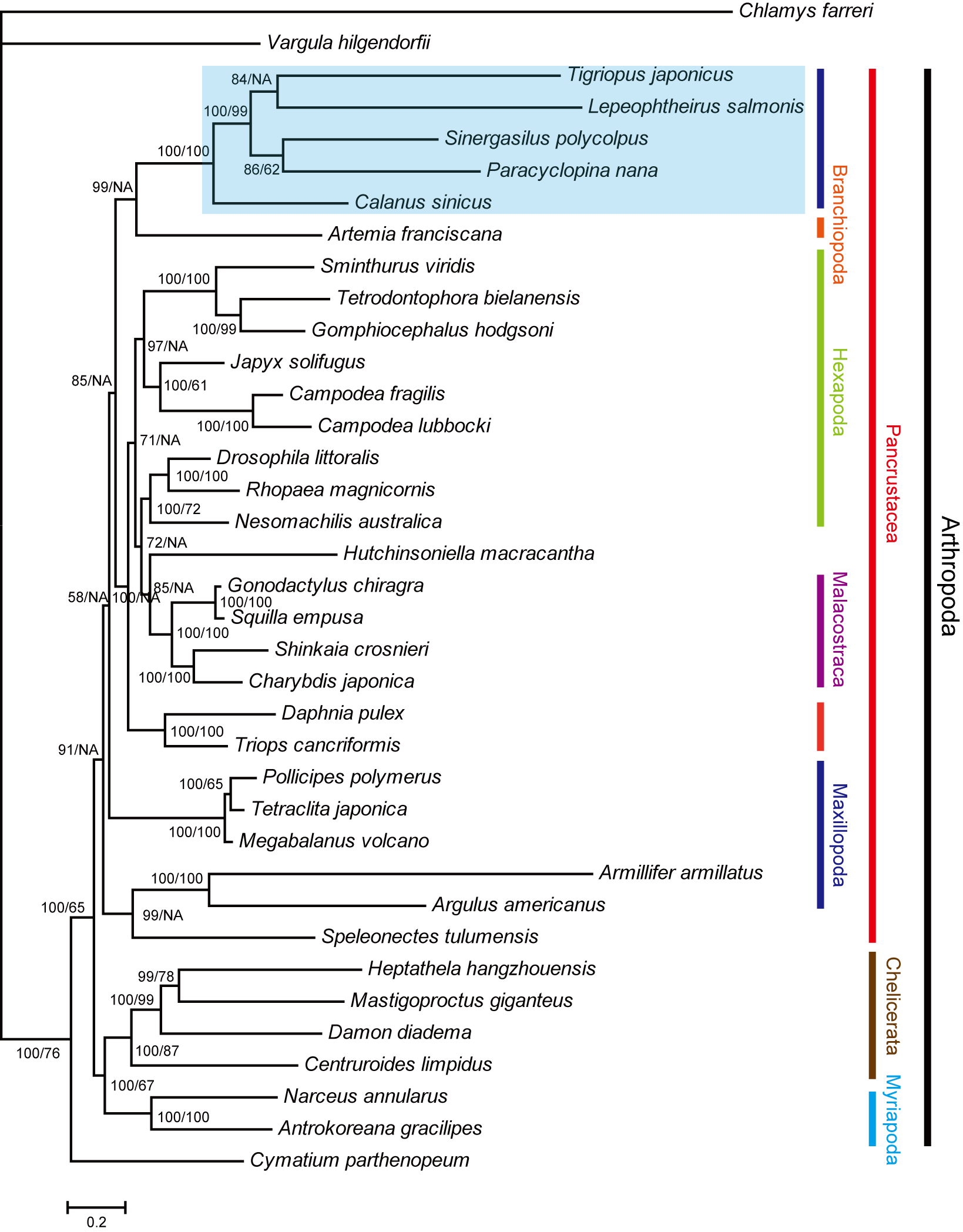

Supplement: Additional file 5 — Phylogenetic tree presenting the monophyly of Copepoda and its position within arthropods inferred from concatenated amino acids of 12 mitochondrial protein coding genes. Tree topologies produced by ML (mtArt model under PHYML) and BI (mtArt model under MrBayes) were compactable. Numbers at the branch nodes refer to Bayesian posterior probabilities and bootstrap support values from left to right. [file 1471-2164-12-73-S5.PNG]

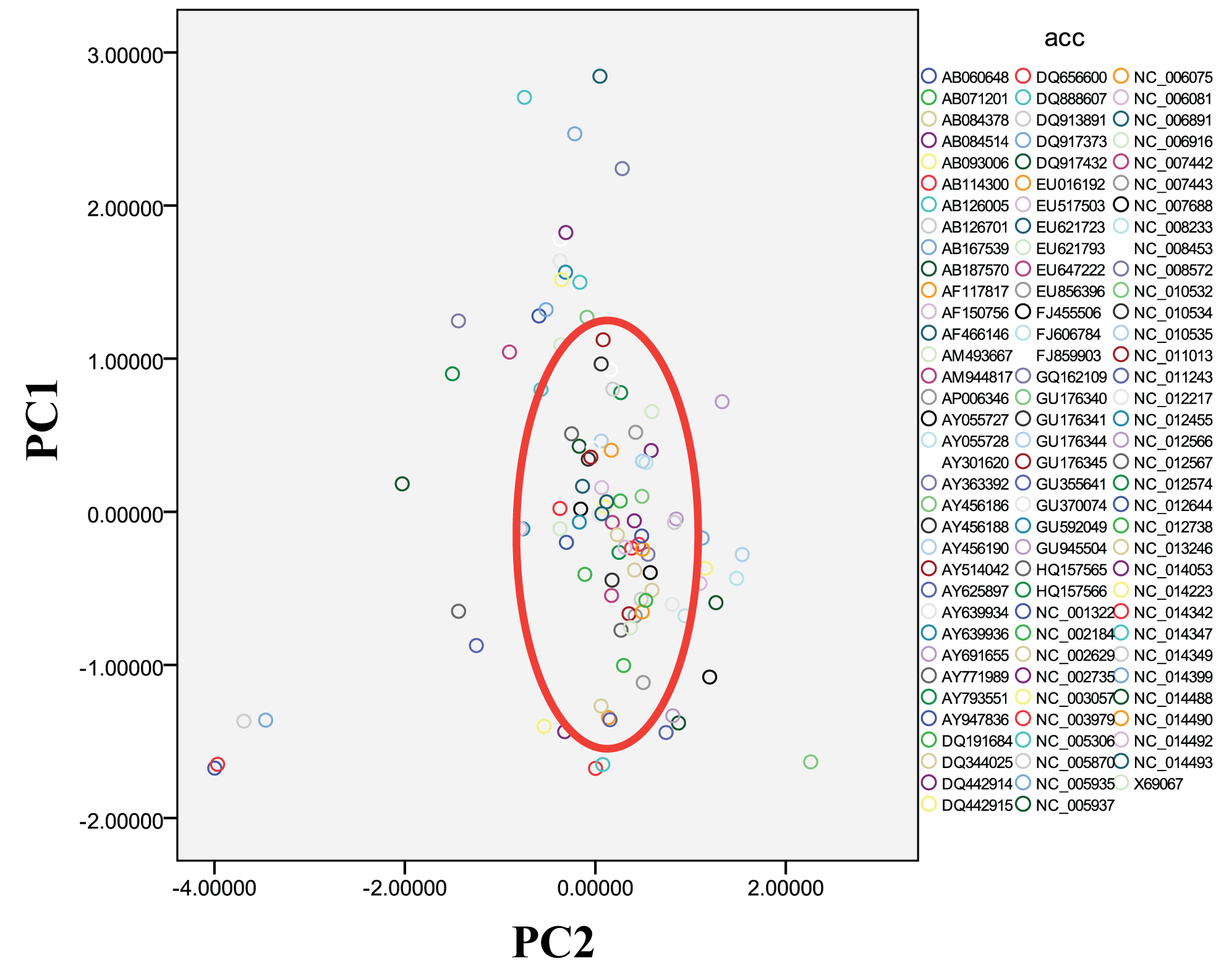

Supplement: Additional file 8 — Nucleotide compositional properties of the candidates for phylogenetic analysis illustrated by principal components analysis (PCA). PCA ordination was based on the proportion of separate nucleotides. PC1 (principal component 1) explained 87% of the total variations and PC2 explained 10% of the total variations. Species were sampled predominantly inside the red ellipse. [file 1471-2164-12-73-S8.PNG]
